# Single-cell transcriptomic landscape of immunometabolism reveals intervention candidates of ascorbate and aldarate metabolism, fatty-acid degradation and PUFA metabolism of T-cell subsets in healthy controls, psoriasis and psoriatic arthritis

**DOI:** 10.3389/fimmu.2023.1179877

**Published:** 2023-07-10

**Authors:** Lu Peng, Ling Chen, Jianji Wan, Wenqi Liu, Shuang Lou, Zhu Shen

**Affiliations:** ^1^ Department of Dermatology, Guangdong Provincial People’s Hospital, Guangdong Academy of Medical Sciences, Southern Medical University, Guangzhou, China; ^2^ Department of Dermatology, Daping Hospital, Army Medical University, Chongqing, China

**Keywords:** TCM, TEM, Treg, immunometabolism, psoriasis, psoriatic arthritis, single-cell transcriptomics

## Abstract

**Introduction:**

The modulation of immunometabolic pathways is emerging as a promising therapeutic target for immune-mediated diseases. However, the immunometabolic features of psoriatic disease and the potential targets for immunometabolic intervention in the different T-cell subsets involved in its pathogenesis remain unclear.

**Methods:**

In this study, we analyzed circulating blood single-cell data from healthy controls (HC), psoriasis (PSO), and psoriatic arthritis (PSA) patients, and revealed their metabolic features of T-cell subsets: CD4+ central memory T cells (TCMs), CD8+ effective memory T cells (TEMs), regulatory T cells (Tregs), mucosal-associated invariant T cells (MAITs ), and γδ T cells. Pearson test was performed to determine the linkages between differential metabolic and inflammatory pathways. Based on these results, we also analyzed the potential impacts of biological antibodies on differential metabolic pathways by comparing the immunometabolism differences between PSA patients without and with biological treatment.

**Results:**

Our results suggest that upregulation of ascorbate and aldarate metabolism, as well as fatty acid degradation, may enhance the immune suppression of Tregs. Enhanced metabolism of alpha-linolenic acid, linoleic acid, and arachidonic acid may inhibit the pro-inflammatory functions of CD4+ TCMs and CD8+ TEMs in PSO and PSA, and protect the immune suppression of Tregs in PSA. We propose that supporting ascorbic acid and fatty acid metabolic pathways may be an adjunctive reprogramming strategy with adalimumab and etanercept therapy.

**Discussion:**

These findings not only provide insights into immunometabolism characteristics of psoriatic disease, but also offer preliminary options for the auxiliary treatment of psoriasis.

## Graphical Abstract

Diagrammatic presentation of data sources, sample grouping, and data analysis procedures.

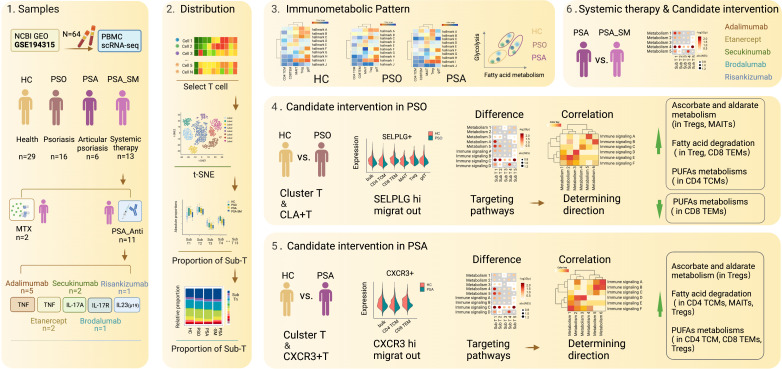

## 1 Introduction

Immunometabolism is not only a process of biosynthesis/catabolism and ATP production, but also its metabolites can act as immune signal molecules. These signal molecules can directly or indirectly affect function/differentiation of immune cells by reprogramming gene transcription and/or post-transcriptional regulation (1, 2). Immunometabolism reprogramming has emerged as one of the major mechanisms central to the physiology and pathology of immune cells (1). It is shown that immune cell subsets with different states/functions utilize distinct metabolic pathways to support survival and functions. For example, activated T cells and quiescent memory T cells mainly rely on glucose catabolism and fatty acids degradation respectively.

Memory T cells bridge innate immunity and acquired immunity, and respond rapidly and powerfully to pathogenic challenges under physiological conditions. However, under pathological or uncontrolled conditions, there is sufficient evidence supporting the involvement of memory T cells in the recurrence of chronic inflammatory disorders, including psoriasis (3). Memory T cells are composed of several subsets according to their functional properties, proliferation and migration potentials: central memory T cells (TCMs), effective memory T cells (TEMs), and tissue-resident memory T cells (TRMs). TCMs have been demonstrated to use intracellular synthetized fatty acids to support fatty-acid oxidation and oxidative phosphorylation (4). Resting TEMs tend to metabolize fatty acids, while glycolysis is started when they are activated to meet the requirements of function transformation (5). TRMs enhance their uptake of extracellular fatty-acids that are abundant in peripheral tissues (e.g. the skin) to maintain their longevity and function (6). In contrast, in regulatory T cells (Tregs) that play pivotal roles in maintaining immune tolerance, forkhead box protein P3 (FoxP3) transcription factor has been shown to promote oxidative phosphorylation and fatty-acid oxidation by enhancing mitochondrial components of electron respiratory complexes (7). Compared with effector T cells, Tregs have less dependence on glycolysis. It can be seen that targeted intervention on immunometabolism is expected to be an important means to manipulate inflammatory reaction. However, disease-intrinsic immunometabolism reprogramming characteristics of different immune cell subsets (e.g. in psoriasis) remain poorly understood.

Psoriasis is an immune-mediated, chronic, recurrent and inflammatory disease leading to physical and psychological burden for individuals and their families (8). Dysfunctional immune T-cell subsets are involved in its development, including TCMs, TEMs, TRMs, Tregs, γδ T cells, and mucosal-associated invariant T cells (MAITs) (9). Immune targeting therapies of biological antibody for TNF-α, IL-12, IL-17 and IL-23 has revolutionized psoriasis development (8). However, the immunometabolic characteristics and the target of reprogramming intervention of psoriasis remain poorly understood. Biological antibodies’ effects on immunometabolism are still unclear.

In this study, we presented the transcriptomic landscape of immune metabolism in circulating T-cell subpopulations in healthy controls (HC), psoriasis (PSO), and psoriatic arthritis (PSA) patients (**Graphical Abstract**), and revealed their immune metabolic reprogramming characteristics. By elucidating the differences in immune metabolism among T-cell subsets and their linkage with immune and metabolic pathways, we showed that upregulating ascorbate and aldarate metabolism and fatty acid degradation may enhance the immune suppression of Tregs in PSO and PSA. Enhancing the metabolism of alpha-linolenic acid, linoleic acid, and arachidonic acid may inhibit the pro-inflammatory function of CD4+ TCMs and CD8+ TEMs in PSO and PSA and protect the immune suppression of Tregs in PSA, thus benefiting the suppression of disease inflammation. Moreover, by comparing the immune metabolism between patients receiving biologic therapy and those not receiving any treatment, we propose that enhancing ascorbate and fatty acid metabolism pathways may serve as friendly reprogramming strategies to assist the treatment of psoriasis with adalimumab and etanercept.

## 2 Materials and methods

### 2.1 Data source

The Single cell RNA sequencing (scRNA-seq) data of peripheral blood mononuclear cells (PBMCs) were downloaded from the GEO repository at the National Center for Biotechnology Information (NCBI) (https://www.ncbi.nlm.nih.gov/geo/query/acc.cgi, GSE194315). All subsequent analyses were performed based on the data from selected 64 subjects, including 29 healthy controls samples (HC), 16 cutaneous psoriasis subjects without systemic medications (PSO), 6 psoriatic arthritis subjects without systemic medications (PSA), and 13 psoriatic arthritis subjects with systemic medications (PSA_SM). According to the different medications used, group PSA_SM was consisted of 2 subjects with methotrexate (MTX) and 11 subjects with targeted biologic antibodies (PSA_Anti), including 2 Secukinumab, 5 Adalimumab, 1 Brodalumab, 1 Risankizumab, and 2 Etanercept (**Graphical Abstract**; **Supplementary Table 1**).

### 2.2 Data processing

The obtained scRNA-seq Matrix File was processed for quality control and cell type annotation. The exact procedure and criteria were given in published articles (10, 11) T cells were selected and further analyzed in subsequent investigation. Visualization and marker genes for each subset were performed using Seurat (version 3.1.1) (12). Marker genes were identified with the threshold parameters: 1. Genes were expressed in more than 25% of cells in the target or control subsets; 2. P value ≤0.01; 3. Log2FC≥0.36. Analysis of differences between two groups was evaluated with two-sided Wilcoxon rank sum tests, and P value <0.05 was considered to be statistically significant.

### 2.3 Subfractions definition

T cells of PSO without/with the expression of *SELPLG* gene (encoding cutaneous lymphocyte-associated antigen, CLA protein) were defined as CLA-/CLA+, and divided into CLA- subfractions and skin-homing CLA+ subfractions. T cells of PSA without/with the expression of *CXCR3* gene were defined as CXCR3-/+, and divided into CXCR3- subfractions and joint chemotactic CXCR3+ subfractions.

### 2.4 Functional enrichment

Gene set variation analysis (GSVA) was performed using GSVA R packages (13). and the latest HALLMARK pathways dataset from the MsigDB with fifty common signaling pathways (14). Gene set enrichment analysis (GSEA) was performed using software GSEA and gene sets from KEGG to identify whether a set of genes shows significant differences in two groups. Briefly, we input gene expression matrix and rank genes by SignaltoNoise normalization method (15). Enrichment scores and p value were calculated with default parameters.

### 2.5 Immunometabolic linkage

Scores of the gene signatures of KEGG gene sets was calculated with the AddModuleScore function in Seurat (version 3.1.1) (12). Pearson test was performed to determine the linkages between gene sets scores of metabolism pathways and immunity pathways.

### 2.6 Data availability statement

The data underlying this article were accessed from the GEO repository at the National Center for Biotechnology Information (NCBI) (https://www.ncbi.nlm.nih.gov/geo/query/acc.cgi, GSE:194315) (10). The derived data generated in this research will be shared on reasonable request to the corresponding author.

## 3 Results

### 3.1 The distribution of T-cell subsets in HC, PSO, PSA, and PSA_SM

**Figure 1:**
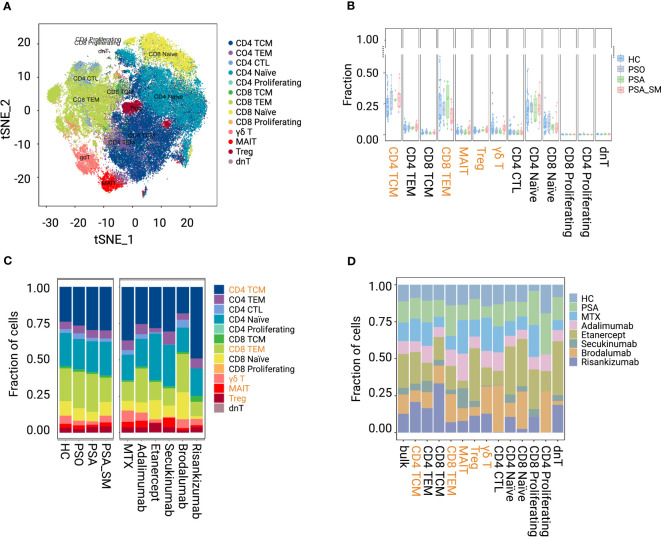
Visualization and proportion of T-cell subsets in group HC, PSO, PSA, PSA_SM. **(A)** tSNE shows the dimensional reduction of T-cell subsets. **(B, C)** Fraction distribution of T-cell subsets in group HC, PSO, PSA, PSA_SM. **(D)** Fraction distribution of T-cell subsets in specific systemic medication groups.

In our investigation, 91,670 T cells were obtained for analyses from the processed data matrix, and were further clustered into 13 subsets: CD4+ TCMs (27.62%), CD4+ TEM (5.43%), CD8+ TCM (1.37%), CD8+ TEMs (21.46%), γδ T cell (4.19%), mucosal-associated invariant T cell (MAIT, 2.39%), Treg (3.51%), CD4+ Naïve (21.78%), CD8+ Naïve (9.05%), CD4+ CTL (2.87%), CD4+ Proliferating (0.05%), CD8+ Proliferating (0.04%) and dnT (0.23%) (**Figure 1A**; **Supplementary Figure 1**). Special attention was paid to the CD4+ TCMs and CD8+ TEMs that we found the largest memory proportion in circulating blood. We also focused on Tregs and two innate immune cell populations, γδ T cells and MAITs (less than those in HC, **Figures 1B, C**).

When comparing PSA_SM with PSA (**Figures 1B, C**), we found that the proportion of CD8+ TEMs in PSA_SM reduced most obviously (reduction rate 37.01%). γδ T cells and MAITs increased by 69.13% and 62.58% in PSA_SM, respectively. Most systemic treatments of PSA_SM resulted in the consistent trend: CD8+ TEMs inhibition and γδ T cell expansion (**Figures 1C, D**). Specifically, brodalumab showed a significant suppression of CD4+ TCMs; Obvious increases of Tregs in Etanercept and MAITs in MTX, adalimumab, secukinumab were observed. These increases may be one of the contributions for their CD8+ TEMs inhibition; The frequency pattern in post-treatment of adalimumab was the closest to that of HC (**Figures 1C, D**).

### 3.2 Immunometabolic patterns of T cell subsets in HC, PSO, and PSA

**Figure 2:**
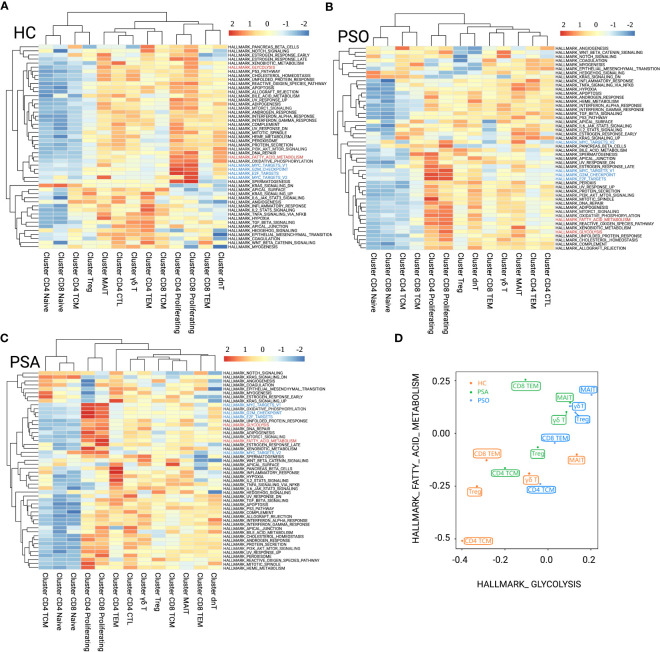
GSVA (HALLMARK) analysis for immunometabolic patterns of T-cell clusters in HC **(A)**, PSO **(B)**, and PSA **(C)**. The focused metabolic gene sets are highlighted in red, and the cycle-related gene sets are highlighted in blue. **(D)** Patterns of fatty acid metabolism and glycolysis gene sets performed among T-cell subsets of group HC, PSO, and PSA based on GSVA scores.

With GSVA (HALLMARK) analysis (**Figures 2A–C**), we found that MAITs and γδ T cells were metabolically active independent of disease status, with enrichment of PI3K/Akt/mTOR signaling, glycolysis, fatty-acid metabolism, and oxidative phosphorylation. In contrast, CD4+ TCMs were naïve-like metabolically inert, with the above-mentioned pathways clearly silenced. CD8+ TEMs were indolence in PSO (**Figure 2B**), and with fatty-acid metabolism preference in PSA (**Figure 2C**). Compared with HC, glycolysis and fatty-acid metabolism were active in PSO Tregs (**Figures 2A, B**). In PSA Tregs, glycolysis was preferred (**Figures 2A, C**). The naïve-like metabolic inertness of CD4+ TCMs and fatty-acid metabolism preference of CD8+ TEMs corresponded to their inhibition of cyclin-related pathways, which just fitted with the published results in which TCMs have better potential of longevity and recall expansion, followed by TEMs (16). Although fatty-acid metabolism and glycolysis of CD4+ TCMs were at an inert baseline compared with other subsets in the same states, their baselines were higher in both PSO and PSA than in HC (**Figure 2D**).

### 3.3 Candidate intervention of immunometabolism in PSO

#### 3.3.1 Immunometabolic differences between PSO and HC

It is known that CLA, encoded by gene *SELPLG*, positive memory T cells distribute in a large number in psoriatic lesions, and CLA is the molecular basis for mediating cell chemotaxis to skin lesions (17, 18). In order to explore the ideal metabolic intervention of immunometabolism reprogramming of PSO, we performed analysis at the level of Cluster_T cells and skin-homing subfractions CLA+T cells, focused on subset of CD4+ TCMs, CD8+ TEMs, Tregs, MAITs, and γδ T cells.

**Figure 3:**
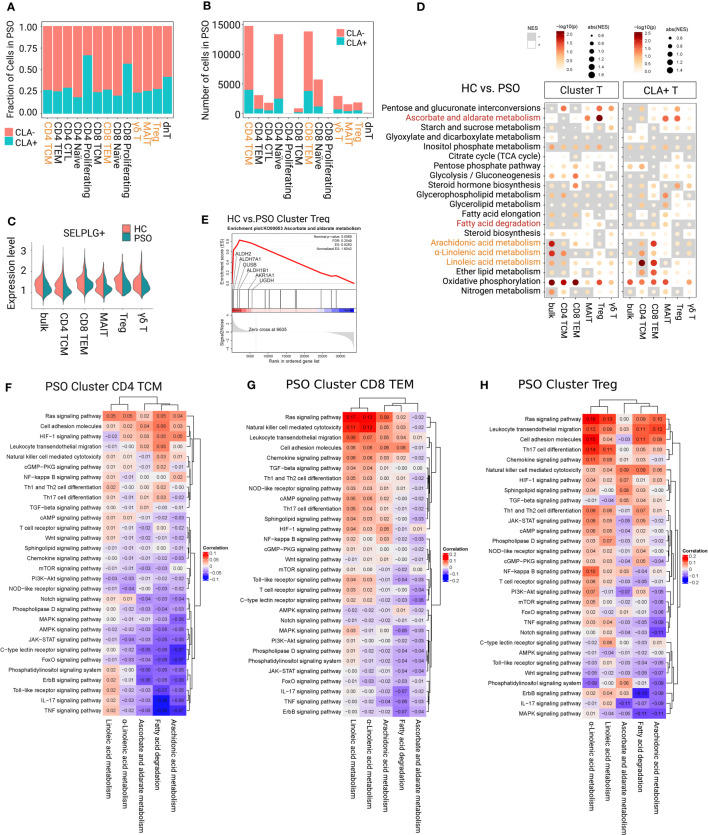
Determining immunometabolic pathways in T-cell subsets of PSO. **(A, B)** Fraction and number of CLA+ and CLA- subfractions. **(C)** The gene expression value of SELPLG showed only a weakly-expressed peak in each focused subset of PSO. **(D)** Metabolic difference landscape of focused subsets between PSO and HC at the levels of Cluster and CLA+ by GSEA. Gray background indicates a negative normalized enrichment score (NES), and the bright background indicates a positive NES. The NES reflects the degree to which a gene set is downregulated (negative NES) or upregulated (positive NES). The area of the dot corresponds to the NES absolute value. The color of the dots corresponds to the P value. **(E)** GSEA showed an obvious increase of ascorbate and aldarate metabolism in Tregs of PSO. Immunometabolic linkage between metabolic pathways of α-linolenic acid metabolism, linoleic acid metabolism, arachidonic acid metabolism, ascorbate and aldarate metabolism, or fatty acid degradation and immune events in CD4+ TCMs **(F)**, CD8+ TEMs **(G)**, and Tregs **(H)** of PSO at the level of Cluster.

Approximately a quarter of the focused subsets expressed *SELPLG* gene (**Figure 3A**), specifically, 27.1% of CD4+ TCMs, 24.71% of CD8+ TEMs, 28.33% of Tregs, 26.1% of MAITs, and 24.16% of γδ T cells in PSO. Among that, CLA+CD4+ TEMs and CLA+ CD8+ TEMs were the dominant in CLA+ subfractions as 27.14% and 25.79% (**Figure 3B**). The gene *SELPLG* showed only a weakly-expressed peak in each focused subsets of PSO (**Figure 3C**), which was different from the two expression peaks in HC. The absence of the upper peak implied that the portion of CLA+T cells with highly expressed *SELPLG* probably have migrated to peripheral skin lesions in PSO. Subsequently, we observed that immune signalings and metabolism pathways of CLA+ subfractions in PSO were generally more active than those of the CLA- subfractions (**Supplementary Figure 2**).

The immunometabolic GSEA of focused subsets between PSO and HC were performed at both level of Cluster_T cells and CLA+T cells (**Figure 3D**). Obviously, downregulation of inositol phosphate metabolism and upregulation of oxidative phosphorylation were observed at both levels in each focused subset in PSO. downregulation of inositol phosphate metabolism and upregulation of oxidative phosphorylation were observed at both levels in each focused subset in PSO. Both level of CD4+ TCMs showed restrained activities in α-linolenic acid, linoleic acid, and arachidonic acid metabolism. CLA+CD8+ TEMs showed an upward trend of the metabolism of three main polyunsaturated fatty acids (PUFAs), and a restrained trend of fatty acid degradation. Tregs and MAITs had an obvious increase in ascorbate and aldarate metabolism at both levels, especially in Cluster Tregs (highlighted in core enrichment genes *ALDH2*, *ALDH7A1*, *UGDH*, and *ALDH1B1*) (**Figures 3D, E**). Cluster Tregs had an enhancement in fatty acid degradation. Both levels of γδ T cells displayed a decrease in α-linolenic acid, and CLA+γδ T cells had a decrease in arachidonic acid metabolism.

#### 3.3.2 Immunometabolic linkage in PSO

On the basis of above differential trends alone, it is difficult to judge whether the observed metabolic changes are protective effects from stress response of disease or concomitant consequences caused by disease attribute. To further identify the intervention direction of the candidate pathways, we analyzed the linkage between candidate metabolic pathways and multiple immune signaling pathways in T-cell subsets of PSO (**Figures 3F–H**; **Supplementary Figures 3**, **4**).

Although oxidative phosphorylation and inositol phosphate metabolism were not ideal metabolic pathways for intervention, we found that the oxidative phosphorylation in focused T-cell subsets of PSO was inversely linked with IL-17 and TNF signaling pathways (**Supplementary Figure 3**). However, oxidative phosphorylation was positively linked with cell adhesion molecules and leukocyte transendothelial migration of CD4+ TCMs and CD8+ TEMs. It is suggested that activating oxidative phosphorylation in PSO may be beneficial to reserve the function of cell adhesion and migration, but not to the secretion of IL-17 and TNF. Inositol phosphate metabolism in CD4+ TCMs and CD8+ TEMs was positively linked with mTOR, chemokine and JAK-STAT signaling pathways, while this linkage was relatively decreased in Tregs.

Among the candidate metabolic pathways in PSO, linoleic acid metabolism was negatively linked with AMPK and PI3-Akt signaling pathways, arachidonic acid metabolism was negatively linked with TNF, MAPK, Jak, and AMPK signaling pathways, and α-linolenic acid metabolism was negatively linked with IL-17, TNF, Jak, PI3-Akt, and NOD-like receptor signaling pathways at both level of CD4+ TCMs (**Figure 3F**; **Supplementary Figure 4A**). The metabolism of three main PUFAs displayed positive linkages with Ras signaling pathway, cell adhesion molecules, leukocyte transendothelial migration, cAMP and chemokine signaling pathway in both level of CD8+ TEMs (**Figure 3G**; **Supplementary Figure 4B**). In addition, arachidonic acid metabolism was positively linked with NF-kappa B signaling pathway in CLA+CD8+ TEMs, fatty acid degradation was negatively linked with proinflammatory IL-17 and TNF signaling pathways in Cluster CD8+ TEMs and CLA+CD8+ TEMs. As indicated, enhancing the main PUFA metabolism pathways may inhibit proinflammatory signals of CD4+ TCMs. Conversely, there was a risk of increasing activation of proinflammatory signaling pathways, cell adhesion and cytotoxicity of CD8+ TEMs. Upregulation of fatty acid degradation could inhibit the proinflammatory pathways in CD8+ TEMs.

In Tregs, (**Figure 3H**; **Supplementary Figure 4C**) ascorbate and aldarate metabolism was observed to be negatively linked with IL-17, PIK-3, and Jak signaling pathways, and positively linked with TGF, HIF-1 signaling pathways. Fatty acid degradation showed a negative linkage with IL-17, MAPK signaling pathways, and a positive linkage with cell adhesion and migration at both levels. In the subfractions of CLA+Tregs, ascorbate and aldarate metabolism showed a stronger linkage with NF-kb, TGF, HIF-1 signaling pathways, cell adhesion molecules and leukocyte transendothelial migration. Fatty acid degradation displayed a stronger linkage with cell adhesion molecules and leukocyte transendothelial migration, and an additional negative linkage with mTOR signaling pathway. Therefore, it could be supposed that strengthening ascorbate and aldarate metabolism and fatty acid degradation would be expected not only to suppress the proinflammatory polarization/plasticity of Tregs, but also to stabilize their immunosuppressive function and promote cell adhesion and migration.

In Cluster MAITs, it was shown that ascorbate and aldarate metabolism was positively linked with mTOR, TGF-β, AMPK and cGMP signaling pathway, and negatively linked with IL-17 and Jak signaling pathways (**Supplementary Figures 4D, E**). In contrast, in the CLA+MAITs, ascorbate and aldarate metabolism had positive linkages with IL-17 and TNF signaling pathways, Th17 and Th1/Th2 cell differentiation. Its linkage with HIF-1 signaling pathway changed from positive to negative. Therefore, upregulation of ascorbate and aldarate metabolism in Cluster MAITs could be expected to enhance their immunosuppressive function and promote their adhesion and migration, but this intervention might promote IL-17-related functions in CLA+MAITs.

For γδ T cells (**Supplementary Figures 4F, G**), arachidonic acid metabolism and fatty acid degradation were negatively linked with IL-17 and TNF signaling pathways at both levels. α-Linolenic acid metabolism was negatively linked with TNF, Jak, AMPK signaling pathways, and cell adhesion molecules at the Cluster γδ T cells, but positively linked with IL-17 signaling pathway in CLA+γδ T cells. Based on these, up-regulations of arachidonic acid metabolism and fatty acid degradation might weaken inflammatory signaling of γδ T cells.

In conclusion, combining the analysis of immunometabolism differences with immunometabolism linkage of T-cell subsets in PSO, we suppose that enhanced ascorbate and aldarate metabolism could stabilize the immunosuppressive population of Tregs and Cluster MAITs. Upregulation of fatty acid degradation could stabilize their immunosuppressive function, and promote adhesion and migration of Tregs, and inhibit the inflammatory signal of CD8+ TEMs. Metabolism ehancement of the three main PUFAs might weaken the proinflammatory signaling and migration of CD4+ TCMs. In contrast, inhibition of PUFAs’ metabolism was detrimental to cell adhesion and cytotoxicity of CD8+ TEMs. Further experiment will be needed to verify these speculations.

### 3.4 Candidate intervention of immunometabolism in PSA

#### 3.4.1 Immunometabolic differences between PSA and HC

**Figure 4:**
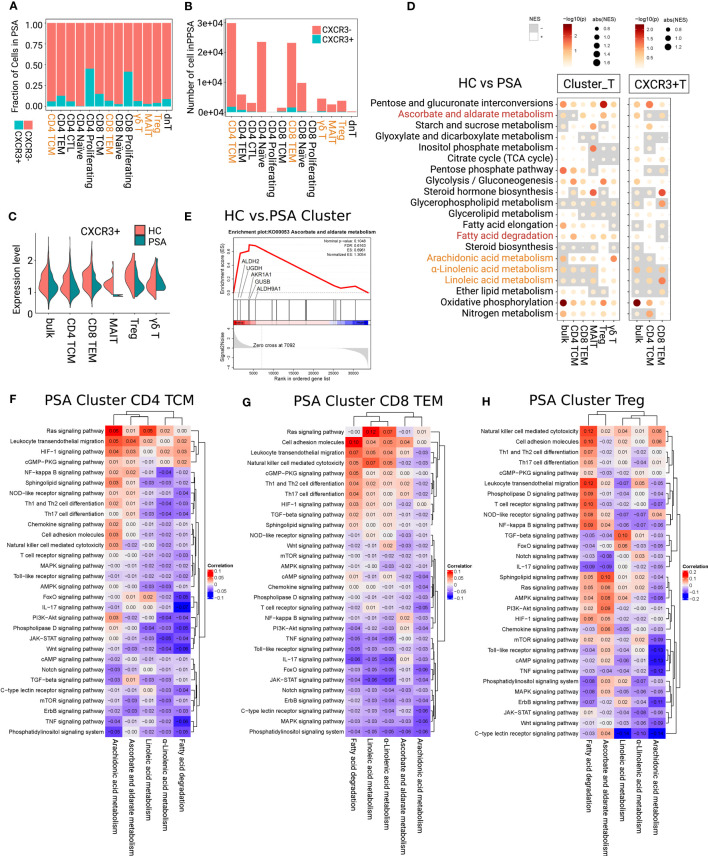
Determining immunometabolic pathways in T cells of PSA. **(A, B)** Fraction and number of CXCR3+ and CXCR3- subfractions. **(C)** The gene expression value of CXCR3 showed loss of high-expressed peak in each focused subsets of PSA. **(D)** Metabolic difference landscape of focused subsets between PSA and HC at the levels of Cluster and CXCR3+ by GSEA. Gray background indicates a negative normalized enrichment score (NES) and the bright background indicates a positive NES. The NES reflects the degree to which a gene set is downregulated (negative NES) or upregulated (positive NES). The area of the dot corresponds to the NES absolute value. The color of the dots corresponds to the P value. **(E)** GSEA showed an obvious increase of ascorbate and aldarate metabolism in Tregs of PSA. Immunometabolic linkage between metabolic pathways of α-linolenic acid metabolism, linoleic acid metabolism, arachidonic acid metabolism, ascorbate and aldarate metabolism, or fatty acid degradation and immune events in CD4+ TCMs **(F)**, CD8+ TEMs **(G)** and Tregs **(H)** of PSA at the level of Cluster.

C-X-C Motif Chemokine Receptor 3 (CXCR3) has been verified to be significantly upregulated on synovial CD8+ T cells. Its ligand, CXCL10, is considered as a predictive biomarker of PSA in patients with psoriasis (9, 19). specifically, 6.13% of CD4+ TCMs, 6.85% of CD8+ TEMs, 4.24% of Tregs, 2.98%of MAITs, and 6.42% of γδ T cells in PSA expressed *CXCR3* gene (**Figures 4A, B**). Similar to CLA+ subfractions in PSO, the expression value of *CXCR3* gene showed only low expression peak in PSA (**Figure 4C**). This was clearly distinguished from that in HC where two expression peaks displayed. Compared with the CXCR3- subfractions in PSA, CXCR3+CD4+ TCMs had a relatively active immunometabolic state (except for the marked silence of ascorbate and aldarate metabolism). The CXCR3+CD8+ TEMs had a relatively silent metabolic state, and a relatively active HIF-1 signaling and glycolysis/gluconeogenesis pathway (**Supplementary Figure 5A, B**).

The immunometabolic GSEA of focused subsets between PSA and HC were performed at both levels of Cluster_T cells and CXCR3+T cells (**Figure 4D**; **Supplementary Figure 5C**). In PSA, oxidative phosphorylation was up-regulated in each focused subset and subfractions. Cluster CD4+ TCMs and Cluster MAITs showed a decrease in α-linolenic acid metabolism and linoleic acid metabolism, and an increase in arachidonic acid metabolism and fatty acid degradation. Arachidonic acid metabolism of CXCR3+CD4+ TCMs were inhibited. Cluster CD8+ TEMs showed the inhibition of α-linolenic acid metabolism and linoleic acid, and the upregulation of arachidonic acid metabolism. CXCR3+CD8+ TEMs showed an increase in metabolism of the three PUFAs and fatty acid degradation. In Cluster Tregs, quite obvious activation of ascorbate and aldarate metabolism (core enrichment genes *ALDH2*, *UGDH*, and *AKR1A1*) (**Figures 4D, E**), and upregulation of fatty acid degradation, α-linolenic acid metabolism, linoleic acid metabolism were observed. Cluster γδ T cells displayed the upregulation of arachidonic acid metabolism and fatty acid degradation.

#### 3.4.2 Immunometabolic linkage in PSA

For further identifying the direction of intervention for the candidate metabolic pathways, we analyzed the linkage between these metabolic pathways and immune signaling pathways in PSA (**Figures 4F–H**; **Supplementary Figures 6**, **7**). The results showed that oxidative phosphorylation was negatively linked with IL-17, TNF, Jak signaling pathways, and positively linked with cell adhesion molecules, and leukocyte transendothelial migration in the focused subsets and subfractions of PSA (**Supplementary Figure 6**).

In CXCR3+CD4+ TCMs (**Supplementary Figure 7A**), α-linolenic acid and linoleic acid metabolism were negatively linked with Th17 cell differentiation and IL-17 signaling pathway, and arachidonic acid metabolism displayed an inverse linkage with TNF signaling pathway and Th17 cell differentiation. Weaker negative linkages between the metabolism of three PUFAs and above immune signaling pathways were observed at the Cluster CD4+ TCMs (**Figure 4F**). Fatty acid degradation was negatively linked with IL-17, TNF signaling pathways and Th17 cell differentiation at the Cluster level. The linkages were strengthened in CXCR3+CD4+ TCMs subfractions, and additional negative linkages with cell adhesion and migration pathways were shown. Taken together, upregulation of fatty acid degradation and PUFA metabolism would be an ideal intervention to intercept the production of inflammatory factors IL-17 and TNF in CD4+ TCMs.

In Cluster CD8+ TEMs (**Figure 4G**), α-linolenic acid and linoleic acid metabolism were negatively linked with IL17, Jak, and TNF signaling pathways, and positively linked with Ras signaling pathway, cell adhesion molecules and leukocyte transendothelial migration. The negative linkages with IL-17 and TNF were enhanced in CXCR3+CD8+ TEMs (**Supplementary Figure 7B**). Arachidonic acid metabolism was negatively linked with most immune signaling pathways in Cluster CD8+ TEMs, while it was turned to be positively linked with TNF, IL-17 and cAMP signaling pathways at the level of CXCR3+CD8+ TEMs. As indicated, enhancing PUFA metabolism may inhibit the proinflammatory signaling of Cluster CD8+ TEMs. However, upregulation of arachidonic acid metabolism may have a risk of activating TNF, IL-17 and cAMP signaling pathways in the small subfractions of CXCR3+ CD8+ TEMs.

For Cluster Tregs (**Figure 4H**), ascorbate and aldarate metabolism was negatively linked with IL-17 and TNF signaling pathways. Fatty acid degradation was negatively linked with IL-17 signaling pathway, and positively linked with leukocyte transendothelial migration. α-Linolenic acid showed negative linkages with Th17 cell differentiation and Jak, NF-kB signaling pathways, and positive linkages with leukocyte transendothelial migration. Linoleic acid was positively linked with TGF-β signaling pathway. Arachidonic acid metabolism was negatively linked with IL-17, TNF, cAMP, and TCR signaling pathway. Therefore, upregulation of ascorbate and aldarate metabolism, fatty acid degradation and three PUFA metabolism could help to stabilize the immunosuppressive function and promote migration of Tregs in PSA.

With regard to Cluster MAITs (**Supplementary Figure 7C**), fatty acid degradation was negatively linked with IL-17, TNF, Jak, cAMP signaling pathways, and Th17 cell differentiation. The metabolism of three PUFAs showed positively linked with cell adhesion molecules and leukocyte transendothelial migration, IL-17 and TNF signaling pathways in MAITs. Based on these, increasing fatty acid degradation and inhibiting the metabolism of three PUFAs could support the inflammatory suppression of MAITs.

In Cluster γδ T cells (**Supplementary Figure 7D**), arachidonic acid metabolism showed negative linkages with IL-17, TNF, HIF signaling pathways, and leukocyte transendothelial migration. The linkages in fatty acid degradation were reversed. Thus, upregulation of arachidonic acid metabolism and inhibition of fatty acid degradation could inhibit the proinflammatory function and migration of γδ T cells.

In summary, combining the analysis of immunometabolism differences with immunometabolism linkage of T-cell subsets in PSA, we supposed that upregulation of ascorbate and aldarate metabolism could stabilize anti-inflammatory function of Tregs. Enhancing fatty acid degradation may suppress the proinflammatory signals and Th17 cell differentiation of CD4+ TCMs and MAITs, and stabilize the anti-inflammatory function and promote cell adhesion and migration of Tregs. Upregulating the metabolism of the three PUFAs might suppress the proinflammatory function of CD4+ TCMs and CD8+ TEMs, and stabilize the anti-inflammatory function of Tregs. These suppositions need further experimental verification.

### 3.5 Effect of systemic medicine on candidate metabolic pathways

**Figure 5:**
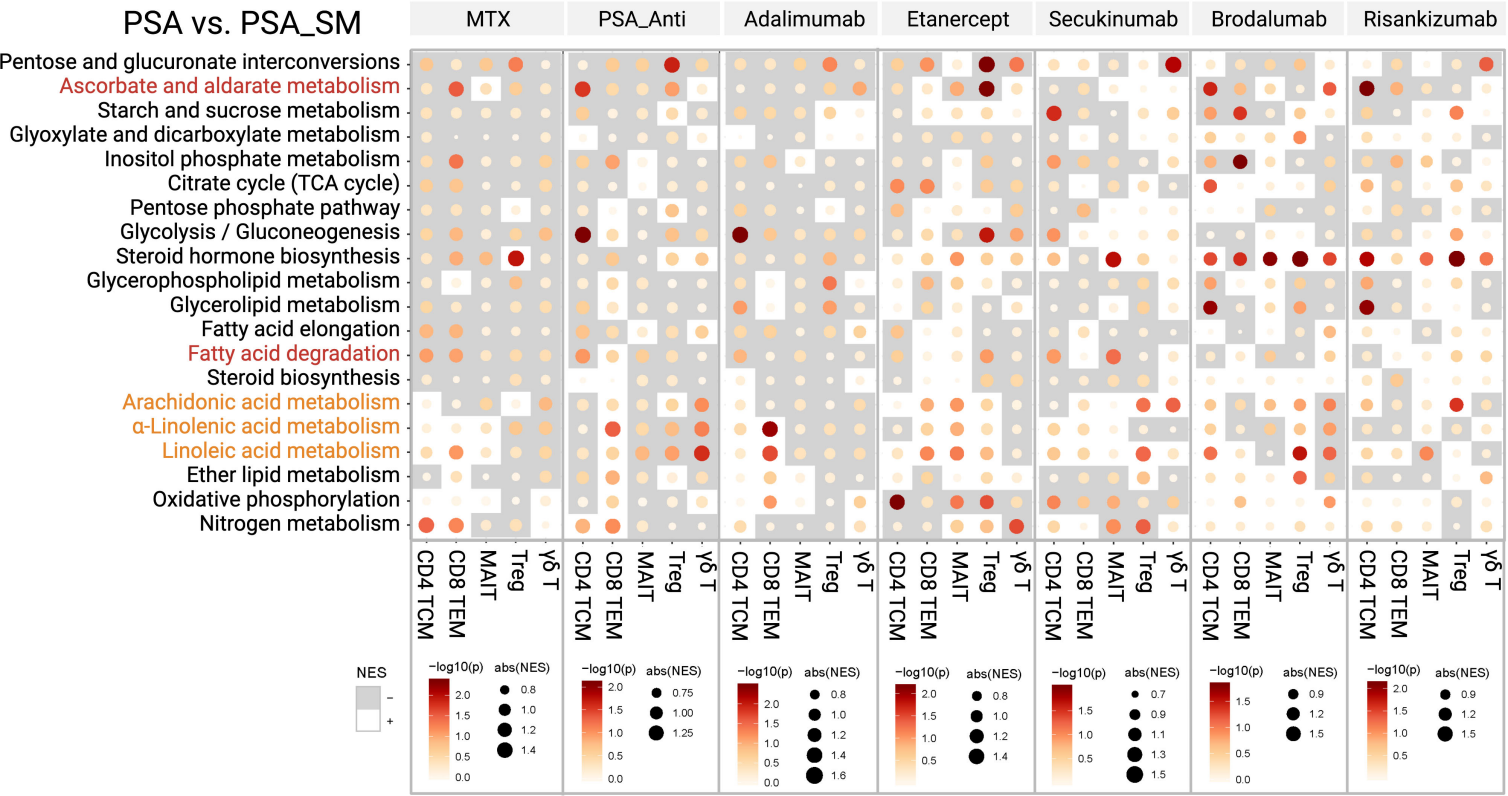
The alterations of candidate immunometabolic pathways in T-cell subsets with systemic medications (PSA_SM).

Finally, GSEA was performed to present the candidate immunometabolic changes in T-cell subsets by systemic therapies (PSA_SM) (**Figure 5**). MTX was found to have the ability to upregulate linoleic acid metabolism of CD4+ TCMs and CD8+ TEMs. The upregulation of fatty acid degradation and the metabolism of three PUFAs was found in CD8+ TEMs by PSA_Anti. Specifically, CD8+ TEMs in etanercept and secukinumab groups and CD4+ TCMs in brodalumab group showed the upregulation of metabolism of the three PUFAs. Ascorbate and aldarate metabolism of Tregs cells and fatty acid degradation of CD4+ TCMs were inhibited in adalimumab and etanercept groups.

## 4 Discussion

In this investigation, we described single-cell transcriptomic landscape of immunometabolism reprogramming in circulating Tregs, CD4+ TCMs and CD8+ TEMs subsets of psoriasis and psoriatic arthritis. We also presented the influences of current biological antibodies on the targeted immunometabolism pathways in psoriasis and psoriatic arthritis.

Here we demonstrated that memory T cell subsets CD4+ TCMs and CD8+ TEMs have the largest proportions in the circulating blood of PSO and PSA, accounting for about 25% of total T cells. CD4+ TCMs have the characteristic of metabolism indolence. CD8+ TEMs were indolence in PSO and fatty acid metabolism preference in HC and PSA. CD8+ TEMs that are capable of immediate effector function decreased significantly after treatments in PSA. Tregs showed different preferences for glycolysis and fatty acids metabolism in PSO and PSA. Compared with HC, these two metabolic demands were simultaneously upregulated in PSO, and glycolysis was preferred in PSA. γδ T cells and MAITs showed similar preferences for glycolysis and fatty acids metabolism. Their proportions in PSO and PSA were less than those in HC group, and rose after PSA treatment. The enrichment patterns of MAITs and Tregs in PSO were similar, and those of γδ T cells and CD8+ TEMs were similar; MAITs and γδ T cells in PSA were similar to CD8+ TCMs, not CD8+ TEMs. These findings suggested MAITs and γδ T cells have distinct degrees and directions of plasticity that maybe due to different immune environments in PSO and PSA. Previous published studies also support this point, that is, MAITs and γδ T cells are multifunctional groups with plasticity, including Treg-like, IL-17/IL-1-producing, or immune-memory phenotype (20–22). Meanwhile, their possible involvements in psoriatic development and their enrichment patterns mentioned above suggest that the functions of circulating MAITs and γδ T cells are likely different from those in peripheral tissues (e.g. the intestine, liver, lung, and psoriasis skin lesions) where they are maintained by local immune environments and mainly IL-17 producing (22–24). The characteristic of plasticity properties of MAITs and γδ T cells, especially immune-memory phenotype, are worthy of comprehensive consideration in psoriatic recurrence investigation.

The observed absence of the upper expression peak of *SELPLG*/*CXCR3* in PSO/PSA implied that the portion of CLA ^high^/CXCR3^high^ T cells may have migrated to local inflammation. Compared with the CLA- subfractions in PSO (**Supplementary Figure 2**), the more active immune signalings and metabolism pathways of CLA+ subfractions suggests that CLA+T cells with weakly-expressed *SELPLG* in the circulating blood may serve as a reserve pool of skin homing. Compared with the CXCR3- subfractions in PSA (**Supplementary Figures 5A, B**), the relatively active immunometabolic state in CXCR3+CD4+ TCMs, as well as the relatively silent metabolic state in CXCR3+CD8+ TEMs suggest that remained CXCR3+CD4+ TCMs were still the preferentially activated subfractions after the migration of CXCR3^high^ population, while the remaining CXCR3+CD8+ TEMs were characteristic by weak pro-inflammation.

To find candidate intervention, we compared immunometabolism pathways between PSO and HC, PSA and HC at the levels of Cluster_T cells and CLA+T/CXCR3+T cells respectively. And the linkage between metabolic and immune signaling pathways was also analyzed. Downregulation of inositol phosphate metabolism and upregulation of oxidative phosphorylation were observed at both levels in each focused subset in PSO. This congruence alteration may be a result of stress response to the disease states and inflammatory pathology. Considering that the above change trends of these two metabolic pathways in pro-inflammatory and anti-inflammatory T-cell subsets are consistent, they are not the suitable candidate targets for metabolism intervention. Noteworthily, we found that ascorbate and aldarate metabolism was significantly upregulated in Tregs in PSO and PSA. In PSO, it was negatively linked with IL-17 and Jak signaling pathways, and positively linked with TGF and HIF-1; In PSA, it was negatively linked with IL-17 and TNF signaling pathways. Both IL-17 and TNF cytokines have been demonstrated to play pivotal roles, and their antagonists are effective treatments for psoriasis (25). In addition, Tregs have the property of inflammation inhibition, therefore, upregulating ascorbate and aldarate metabolism seems to be a promising candidate target to enhance psoriasis treatment.

The immunometabolism differences between PSO and HC, PSA and HC at the levels of Cluster_T cells and CLA+T/CXCR3+T cells respectively combining with the linkage between metabolic and immune signaling pathways were analyzed. we supposed that upregulation of ascorbate and aldarate metabolism as well as fatty acid degradation may enhance the immune suppression of Tregs in PSO and PSA. Enhanced metabolism of alpha-linolenic acid, linoleic acid, and arachidonic acid may inhibit the pro-inflammatory functions of CD4+ TCMs and CD8+ TEMs in PSO and PSA and protect the immune suppression of Tregs in PSA.

Ascorbate and aldarate metabolism has close relationship with glucuronate pathway. The glucuronic acid produced by the latter can be used to synthesize glycosaminoglycans (such as heparin, hyaluronic acid, chondroitin sulfate), and also provide ascorbic acid (vitamin C) to participate in ascorbate and aldarate metabolism (26). Molecules with obvious changes in our investigation in ascorbate and aldarate metabolism mainly included ALDH2, ALDH7A1, ALDH1B1, AKR1A1, GUSB, and UGDH (**Figures 2E**, **5E**). The latter two, UGDH and GUSB also participate in biosynthesis and degradation of glycosaminoglycans respectively. Heparan sulfate is a kind of glycosaminoglycans, and acts at extracellular-matrix interface to modulate cell signaling. Its mimetic PG545 (pixatimod) has been indicated as a potent inhibitor of Th1/Th17 effector functions and inducer of FoxP3+ Tregs (27).

ALDHs that were found to be differentially expressed in ascorbate and aldarate metabolism are family members of aldehydedehydrogenase. They (e.g. AKR1A1) can reduce the aldehydes produced by alcohol metabolism and lipid peroxidation into alcohols to play the detoxification function. It is speculated that maintaining immunosuppressive ability of Tregs during cyclophosphamide treatment of graft-versus-host disease was partly attributed to the detoxification ability of highly expressed ALDH1 (28). In addition, ALDHs are cytosolic enzymes that catalyze the conversion of retinal to retinoic acid (29). Retinoic acid can function with TGF-β to promote conversion of naive T cells into FoxP3+ Tregs and also sustain thymus-derived Treg stability under inflammatory conditions, thereby, maintain immune tolerance (30, 31).

Ascorbate and aldarate metabolism is a vital carbohydrate metabolic pathway, and can protect cells from oxidative damages. Ascorbate is the anion of ascorbic acid (vitamin C), an organic compound with strong antioxidant properties. Vitamin C has been demonstrated to promote the differentiation of peripheral blood CD4+FoxP3-T cells into FoxP3+Tregs *in vivo*. In addition, it facilitates demethylation of FoxP3 enhancer to stabilize Treg’s immunosuppressive function (32–34). An ascorbic acid derivative has been shown to ameliorate imiquimod-induced psoriasis-like dermatitis by suppressing inflammatory cytokine (IL-1β and TNF-α) (35). Furthermore, the introduction of antioxidants such as vitamin C in diet therapy is recommended for persons with psoriasis (36).

With regard to fatty acid degradation, its primary process is the β-oxidation and the release of acetyl-CoA. Like glycolysis traditionally engaged by effector T cells, fatty acid degradation has also been proved to be an important metabolic pathway for the survival and function of immune cells. It has been proposed that FoxP3+ Tregs and CD8+ memory T cells mainly rely on the degradation of long-chain fatty acids (e.g. palmitate oxidation) to meet their energy needs (37, 38). In addition to circulating blood immune cells, survival of tissue-resident memory T cells in psoriatic lesions have also been confirmed to require free fatty-acid uptake and metabolism (6). In our study, we found that upregulation of fatty acid degradation could stabilize immunosuppressive functions of Tregs and promote their adhesion and migration in PSO and PSA; At the same time, its upregulation could inhibit proinflammatory signals of CD8+ TEMs in PSO, CD4+ TCMs and MAITs in PSA.

According to different structures, fatty acids can be divided into saturated fatty acids and unsaturated ones, and the latter can be further divided into monounsaturated fatty acids and polyunsaturated fatty acids (PUFAs) (39). Close conversion and connection exist among PUFAs. PUFAs, including α-linolenic acid (omega-3, ω-3), linoleic acid (ω-6) and arachidonic acid (AA, ω-6), play roles in various chronic inflammatory and autoimmune diseases through various mechanisms, including gene expression, cellular metabolism, and signal transduction (40). AA can be produced from linoleic acid by carbon-chain elongation enzyme and dehydrogenase. α-Linolenic acid can generate high unsaturated fatty acids (HUFAs) such as eicosapentaenoic acid (EPA) and docosahexaenoic acid (DHA). HUFAs, in the form of membrane phospholipids, are hydrolyzed by phospholipase A2 enzymes, releasing lysophospholipids and free fatty acids. Free AA, EPA, and DHA can produce eicosanoids under the action of lipoxygenase and cyclooxygenase. These eicosanoids include prostaglandins (PGs), thromboxanes, and leukotrienes (LTs). They play an important role in the regulation and maintenance of inflammation and hemodynamics, and mediate inflammatory and allergic disorders, including psoriasis and atopic dermatitis (41, 42). Because PGE2, the derivative of AA, is involved in the initiation of inflammation and immune response, the derivative of AA is generally considered to be an inflammatory promoter, but this is not completely the case. It has been shown that PGE2 can restrain the productions of interleukin-6 (IL-6) and TNF-α, and trigger lipoxin A4 production from AA to initiate inflammation resolution (43). In addition, AA can also produce lipoxygenin with anti-inflammatory effect under the catalysis of 15- lipoxygenase. EPA and DHA are generally considered to play anti-inflammatory roles in the body. Firstly, EPA gives rise to eicosanoids that often have lower pro-inflammatory potency than those produced from AA. Next, EPA and DHA can promote expressions of inflammation-resolving mediators of resolvins and protectins (44). In addition, EPA and DHA can constrict the activation of pro-inflammatory transcription factor nuclear factor κB, and enhance transcription factor peroxisome proliferator-activated receptor γ (PPARγ), leading to anti-inflammatory effects (e.g. strengthening Treg responses by upregulation of fatty acid oxidation) (45).

The degradation (β-oxidation) of PUFAs have been suggested to improve immune maturation/function and modulate systemic inflammation (46–48). In our investigation, PUFA metabolisms were inhibited in CD4+ TCMs of PSO at the levels of Cluster_T cells and CLA+T cells respectively. α-Linolenic acid and linoleic acid metabolism were inhibited in cells of CD4+ TCMs and CD8+ TEMs, and activated in Tregs of PSA. Therefore, regulation of the metabolism of PUFAs is supposed to be a candidate target of immunometabolism reprogramming in psoriatic disease.

Compared with those of α-linolenic acid and linoleic acid, effects of AA on psoriatic development and T-cell subsets are extensively studied. Abnormal AA metabolism was one of the mainly disturbed metabolic pathways in psoriasis (49, 50). It has been shown that stimulation of AA-regulated calcium-selective channel on T cells induced not only a calcium-influx, but also phosphorylation of components of T cell receptor signaling cascade, leading to synovial inflammation in a mouse model (51). PGE2, the derivative of AA, is proven to exert dual roles in mouse experimental autoimmune encephalomyelitis, facilitating Th1 and Th17 cell generation redundantly through PGE receptor 4 (EP4) and EP2, meanwhile, weakening the invasion of these T cells into the brain by guarding blood-brain barrier through EP4 (52). In a mouse model of dermatitis, T cell-intrinsic EP2/EP4 signaling triggered by PGE2 was suggested to be critical in IL-23-driven generation of pathogenic Th17 cells (53). However, the research conclusions in this regard are not completely consistent. Other studies showed that prostaglandin subtypes metabolized from AA (PGE2), dihomo-gamma-linolenic acid (PGE1) and EPA (PGE3) significantly decreased Th1 cytokine production (e.g. IFN-γ) resulting in a shift of the Th1/Th2 balance toward Th2 dominance in circulating blood (54, 55). In addition, PGE2 was shown to inhibit the proliferation of CD4+ tissue memory T cells through EP4 receptor after inflammation subsided in a human model of acute inflammation driven by intradermal UV-killed *Escherichia coli* (56). Meanwhile, PGD2, another derivative of AA, was also suggested to promote inflammation resolution through PGD2/DP1 axis (57).

Linoleic acid is an essential fatty acid for the human body. It has been considered as a dietary supplement and to play important roles in lowering cholesterol, intellectual development, and repairing barriers. In a multicenter, randomized, controlled trial, a moisturizer containing linoleic acid-ceramide was shown to enhance therapeutic effects of topical glucocorticoids in psoriasis and delay psoriatic relapse (58). Downregulated linoleic acid in plasma is observed in gingivitis patients and is connected to IL-17, TGF-β and IL-10 signalings, which are related closely to Th17 and Treg pathways (59). Similarly, conjugated linoleic acid (a group of positional and geometric isomers of linoleic acid) was demonstrated to increase the expression of IL-10, TGF-β, and inducible nitric oxide synthase in a mouse model of giardiasis infection (60). In a mouse mammary tumor model, when taken up by T cells, linoleic acid was shown to promote naïve T-cell apoptosis and inhibit TNF-α production. Mechanically, linoleic acid induced increased mitochondrial reactive oxygen species (ROS) and epidermal fatty acid binding protein (E-FABP) productions in T cells, and the latter is vital in facilitating linoleic acid mitochondrial transport and cardiolipin incorporation (61). Taken together, it tends to suggest that the intake of linoleic acid may have therapeutic efficacy against cellular autoimmune disorders, especially type 1 immune response characterized by overproduction of IFN-γ and TNF-α, e.g. in psoriasis. On the other hand, excessive intake of linoleic acid may aggravate type 2 immune response as seen in atopic dermatitis with IL-4 and IL-13 overproduction (62).

α-Linolenic acid, an essential omega-3 fatty acid widely found in plant seed oils and beans, has been reported to have cardiovascular-protective, neuro-protective, anti-inflammatory, and antioxidative effects (63). α-Linolenic acid is the precursor of longer chain omega-3 fatty acids, EPA and DHA. Its effects on T cells are mainly studied through EPA and DHA. Numerous studies have been conducted to supplement EPA and DHA with fish oil intake, so as to increase the proportion of EPA&DHA/AA. EPA and DHA can reduce the number of activated T cells in PBMCs of healthy subjects in a dose-dependent manner (64). They can also reduce ROS and proinflammatory cytokines of IL-1, TNF- α and IL-6, regulate Th1/Th2 Imbalance, and decrease the expression of some adhesion molecules (40, 65). Obesity is known as a risk factor for incidence and aggravation of psoriasis (66). It was previously demonstrated that adipose-derived stem cells from obese, but not lean adipose tissue promoted IL-17A-producing Th17 cells, and α-linolenic acid could reverse this process (67). This immunometabolic mechanism may help to explain the beneficial effects of PUFAs in IL17A-related inflammatory disorders. In addition to the abnormal functions of effector T cells in psoriasis, Tregs are impaired in their suppressive function leading to an altered Th17/Treg balance (68). DHA-primed dendritic cells have been reported to attenuate T-cell proliferation and increase T-cell proportion with expression phenotype of CD4+FoxP3+CTLA-4+, CD4+FoxP3+Helios+ or CD4+FoxP3+PD-1+ (69). AA-primed dendritic cells have a similar effect, which indicates these fatty acids can promote induction of Tregs.

The application of PUFAs in psoriasis has been examined in numerous studies, however, their outcomes were sometimes opposing and inconclusive (70, 71). The possible reasons are insufficient supplement dosage and no restriction on the intake of saturated fatty acids. A meta-analysis involved 560 patients assessing the efficacy of ω-3 fatty acids in treating psoriasis supported high dosage (1800 mg/day EPA) of ω-3 PUFA supplementation for the improvement of clinical parameters (e.g. erythema, itching, scale, or joint pain) (72). In addition, supplementary treatments with ω-3 fatty acids complemented UVB, etretinate, and topical tacalcitol in psoriasis, and made a significant contribution to reducing PASI (Psoriasis Area and Severity Index) score and improving DLQI (Dermatology Life Quality Index) (73–75). These data suggest that combination regimen with PUFAs is likely to improve the treatment and management of psoriasis.

In addition, we presented the immunometabolic changes of T-cell subsets in patients with systemic therapies (PSA_SM). We showed that systemic therapies, including biological antibodies (e.g. adalimumab, secukinumab, risankizumab, etc.) could influence some immunometabolic events of circulating T cells in PSA patients, mainly focusing on increasing linoleic acid and α-linolenic acid metabolisms of CD4+ TCMs and CD8+ TEMs. In fact, the metabolic effects of most biological antibodies on T cells are limited to certain immunometabolic pathways. Moreover, this effect is not so “perfect” in some subpopulations. For example, adalimumab commonly used for PSA could increase α-linolenic acid metabolism in CD8+ TEMs, but reduce fatty acid degradation in CD4+ TCMs and ascorbate and aldarate metabolism in Tregs. However, it is just these “imperfections” that provide space for optimization of therapeutic schedule in psoriatic diseases. The differential analysis of these immunometabolic pathways of T-cell subsets is not only expected to improve the understanding of pathogenesis of psoriasis and psoriatic arthritis, but also to provide a possible explanation for the low response, drug resistance and secondary failure of biological antibody therapy in certain patients. Therefore, given the close relationship between T cells and metabolic pathways, it suggests that development of targeted interventions based on our findings will hopefully provide ideal adjunctive therapies for biological antibody treatments in psoriatic diseases.

There are several limitations in our study. Firstly, samples treated with biological antibodies only come from PSA, not from PSO patients. However, considering that there is a large overlap and sharing of biological antibodies for the treatment of the two diseases, we can also see the impact of these biological antibodies on immunometabolic reprogramming through the comparison between PSA_SM and PSA. In the following investigation, we will increase the sample size with systematic treatments, and conduct more detailed analysis in combination with factors such as drug dose and course of treatment. Secondly, the circulating T-cell subsets are considered as the home base or reserve forces for inflammatory cells at the skin, and further study of immunometabolic characteristics of local inflammatory-cell subsets will be a supplement and support to our results. Finally, our conclusions need to be further confirmed by experiments *in vitro*.

Collectively, we analyzed and presented the transcriptomic landscape of immunometabolism in circulating CD4+ TCMs, CD8+ TEMs, Treg, MAITs, γδ T-cell subsets based on the data of 64 samples of HC, PSO, PSA and PSA_SM from GEO database. According to immunometabolism characteristics of T-cell subsets, we identified double indolence of glycolysis and fatty acid metabolism in CD4+ TCMs, fatty acid metabolism dependence in CD8+ TEMs of HC and PSA, and immunometabolism plasticity in Tregs. In addition, we identified the candidate intervention targets for the immunometabolic reprogramming of T-cell subsets in PSO and PSA according to the difference of immunometabolism in groups and the linkage between immune and metabolic pathways. Our analyses suggest that upregulation of ascorbate and alkaline metabolism could stabilize immunosuppressive function of Treg cells in PSO & PSA and MAITs in PSO, and promote their migration. Upregulation of fatty acid degradation could stabilize Treg immunosuppressive function in PSO and PSA, promote their adhesion and migration, and meanwhile inhibit proinflammatory signal pathways of CLA+CD8+ TEMs in PSO, MAITs and CD4+ TCMs in PSA. Enhancing the metabolism of three main PUFAs (α-linolenic acid, linoleic acid, and arachidonic acid) could inhibit pro-inflammatory functions of CLA+CD4+ TCMs in PSO, Cluster CD4+ TCMs and CD8+ TEMs in PSA, and also stabilize the immune-suppression of Tregs in PSA. The identification and analysis of the transcriptomic landscape of immunometabolism in T cell subsets is not only hopeful to further understand the pathogenesis and development mechanisms, but also will provide promising candidates of adjunctive therapies for psoriatic diseases.

## Data availability statement

The data underlying this article were accessed from the GEO repository at the National Center for Biotechnology Information (NCBI) (https://www.ncbi.nlm.nih.gov/geo/query/acc.cgi, GSE194315).

## Ethics statement

Ethical review and approval was not required for the study on human participants in accordance with the local legislation and institutional requirements. Written informed consent for participation was not required for this study in accordance with the national legislation and the institutional requirements.

## Author contributions

Conceptualization: LP, ZS; Data Curation: LP; Formal Analysis: LP; Funding Acquisition: ZS; Investigation: LC, JW; Methodology: LP, LC, JW, ZS; Project Administration: LC, ZS; Resources: ZS. JW; Software: LP; Supervision: ZS; Validation: LC, ZS; Visualization: LP, SL, WL; Writing - Original Draft Preparation: LP, LC, ZS; Writing - Review and Editing: LP, LC, ZS. All authors contributed to the article and approved the submitted version.

## Supplementary material

Supplementary Figure 1 Heatmap clustering of the differential genes (Top5) within T-cell subsets from group HC, PSO, PSA, and PSA_SM.

Supplementary Figure 2 Immunometabolic difference landscape of T cells within PSO, and between PSO and HC. **(A, B)** Immunometabolic difference landscape between subfractions of CLA-T cells and CLA+T cells in PSO by GSEA. **(C)** Immune signaling difference landscape of focused subsets between PSO and HC at the levels of Cluster and CLA+ by GSEA. Gray background indicates a negative normalized enrichment score (NES) and the bright background indicates a positive NES. The NES reflects the degree to which a gene set is downregulated (negative NES) or upregulated (positive NES). The area of the dot corresponds to the NES absolute value. The color of the dots corresponds to the P value.

Supplementary Figure 3 Immunometabolic linkage between metabolic pathways of inositol phosphate metabolism, glycerolipid metabolism, glycerophospholipid metabolism, glycolysis/gluconeogenesis, pentose phosphate pathway, citrate cycle (TCA cycle), or oxidative phosphorylation and immune events in T cells of PSO. Immunometabolic linkage in CD4+ TCMs **(A, B)**, CD8+ TEMs **(C, D)**, Tregs **(E, F)**, MAITs **(G, H)**, γδ T cells **(I, J)** of PSO at the levels of Cluster and CLA+.

Supplementary Figure 4 Immunometabolic linkage between metabolic pathways of α-linolenic acid metabolism, linoleic acid metabolism, arachidonic acid metabolism, ascorbate and aldarate metabolism, or fatty-acid degradation and immune events in CD4+ TCMs **(A)**, CD8+ TEMs **(B)**, Tregs **(C)** of PSO at the level of CLA+, and MAITs **(D, E)**, γδ T cells **(F, G)** of PSO at the levels of Cluster and CLA+.

Supplementary Figure 5 Immunometabolic difference landscape of T cells within PSA, and between PSA and HC. **(A, B)** Immunometabolic difference landscape between subfractions of CXCR3-T cells and CXCR3+T cells in PSA by GSEA. **(C)** Immune signaling difference landscape of focused subsets between PSA and HC at the levels of Cluster and CXCR3+ by GSEA. Gray background indicates a negative normalized enrichment score (NES) and the bright background indicates a positive NES. The NES reflects the degree to which a gene set is downregulated (negative NES) or upregulated (positive NES). The area of the dot corresponds to the NES absolute value. The color of the dots corresponds to the P value.

Supplementary Figure 6 Immunometabolic linkage between metabolic pathways of inositol phosphate metabolism, glycerolipid metabolism, glycerophospholipid metabolism, glycolysis/gluconeogenesis, pentose phosphate pathway, citrate cycle (TCA cycle), or oxidative phosphorylation and immune events in CD4+ TCMs **(A, B)**, CD8+ TEMs **(C, D)** of PSA at the levels of Cluster and CXCR3+, and Tregs **(E)**, MAITs **(F)**, γδ T cells **(G)** of PSA at the level of Cluster.

Supplementary Figure 7 Immunometabolic lingkage between metabolic pathways of α-linolenic acid metabolism, linoleic acid metabolism, arachidonic acid metabolism, ascorbate and aldarate metabolism, or fatty-acid degradation and immune events in T cells of PSA. Immunometabolic lingkage in CD4+ TCMs **(A)** and CD8+ TEMs **(B)** of PSA at the level of CXCR3+, and MAITs **(C)**, γδ T cells **(D)** of PSA at the level of Cluster.
