# Supplementary figures and images for: Single-cell transcriptomic landscape of immunometabolism reveals intervention candidates of ascorbate and aldarate metabolism, fatty-acid degradation and PUFA metabolism of T-cell subsets in healthy controls, psoriasis and psoriatic arthritis
Source: Front Immunol. 2023 Jul 10;14:1179877. doi: 10.3389/fimmu.2023.1179877 (PMC10363747; doi:10.3389/fimmu.2023.1179877)

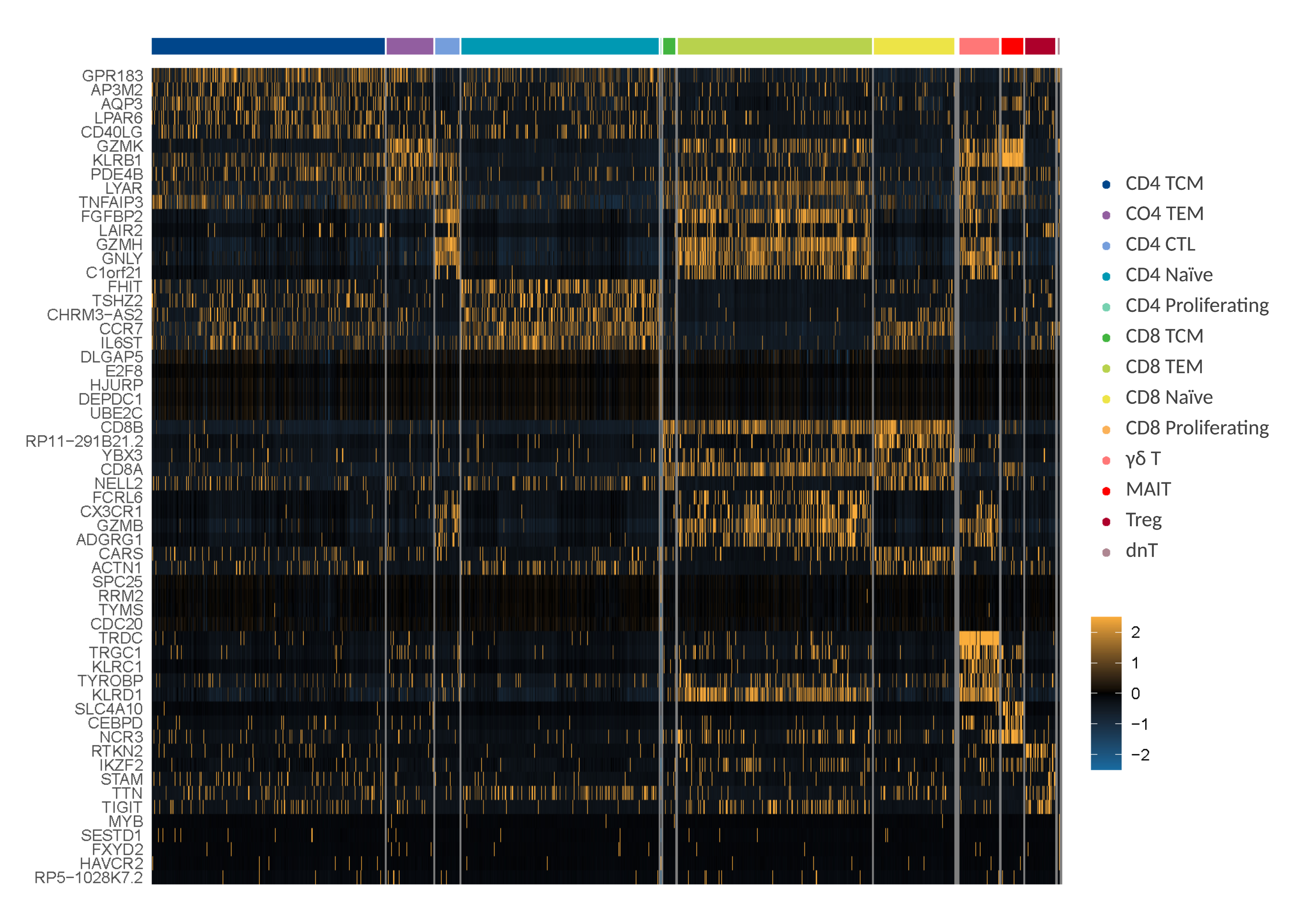

Supplement: Supplementary Figure 1 — Heatmap clustering of the differential genes (Top5) within T-cell subsets from group HC, PSO, PSA, and PSA_SM. [file Image_1.jpeg]

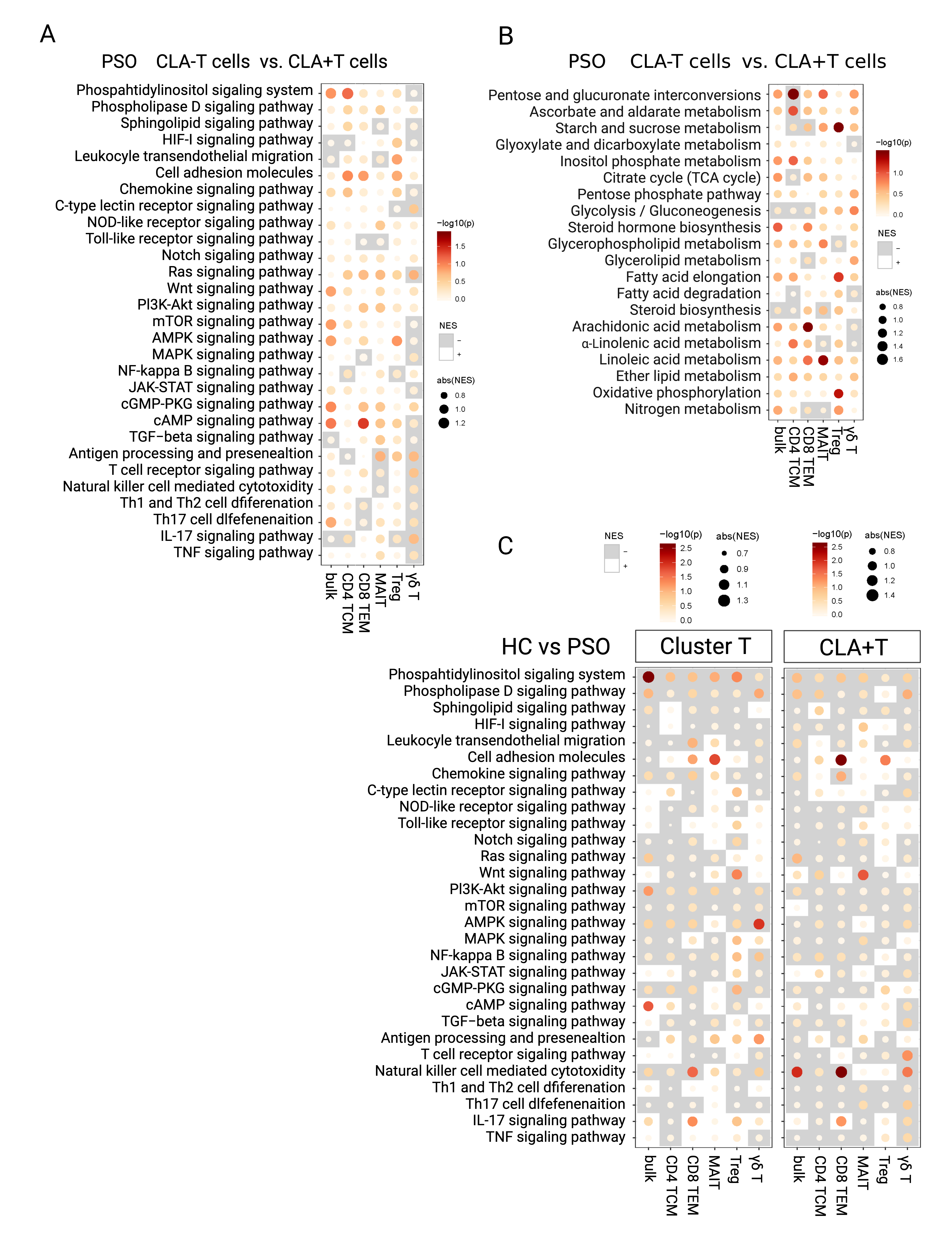

Supplement: Supplementary Figure 2 — Immunometabolic difference landscape of T cells within PSO, and between PSO and HC. (A, B) Immunometabolic difference landscape between subfractions of CLA-T cells and CLA+T cells in PSO by GSEA. (C) Immune signaling difference landscape of focused subsets between PSO and HC at the levels of Cluster and CLA+ by GSEA. Gray background indicates a negative normalized enrichment score (NES) and the bright background indicates a positive NES. The NES reflects the degree to which a gene set is downregulated (negative NES) or upregulated (positive NES). The area of the dot corresponds to the NES absolute value. The color of the dots corresponds to the P value. [file Image_2.jpeg]

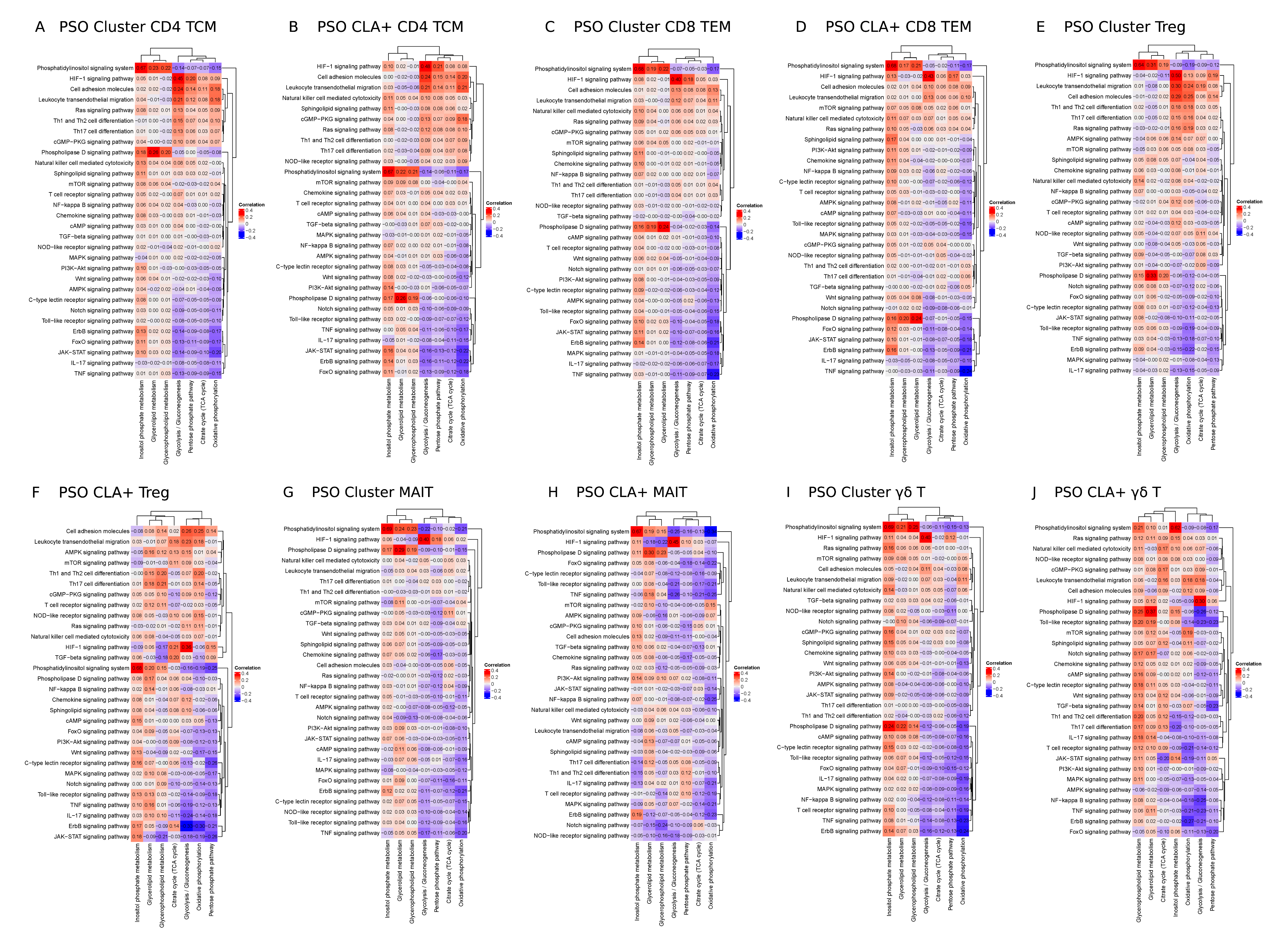

Supplement: Supplementary Figure 3 — Immunometabolic linkage between metabolic pathways of inositol phosphate metabolism, glycerolipid metabolism, glycerophospholipid metabolism, glycolysis/gluconeogenesis, pentose phosphate pathway, citrate cycle (TCA cycle), or oxidative phosphorylation and immune events in T cells of PSO. Immunometabolic linkage in CD4+ TCMs (A, B), CD8+ TEMs (C, D), Tregs (E, F), MAITs (G, H), γδ T cells (I, J) of PSO at the levels of Cluster and CLA+. [file Image_3.jpeg]

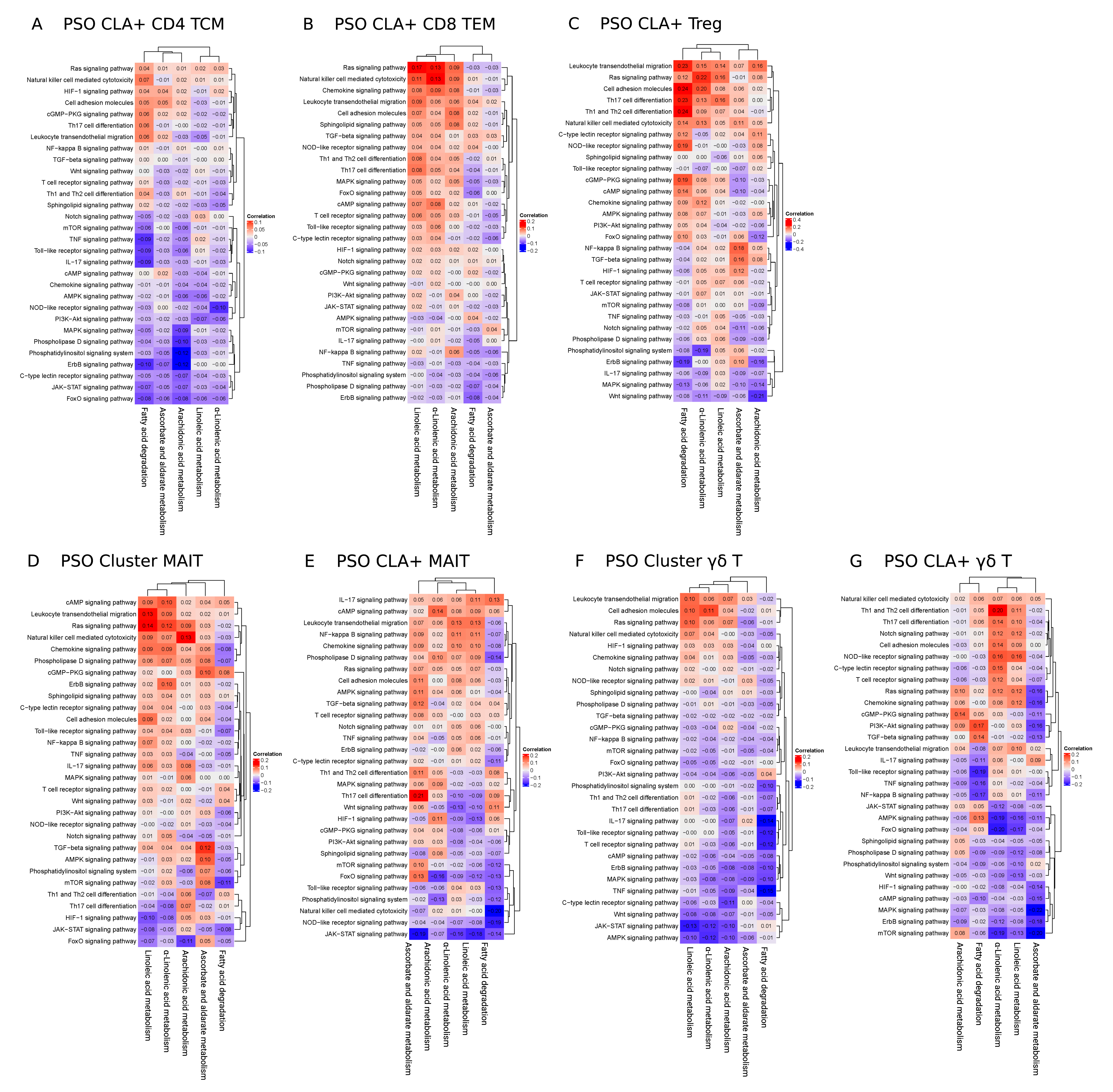

Supplement: Supplementary Figure 4 — Immunometabolic linkage between metabolic pathways of α-linolenic acid metabolism, linoleic acid metabolism, arachidonic acid metabolism, ascorbate and aldarate metabolism, or fatty-acid degradation and immune events in CD4+ TCMs (A), CD8+ TEMs (B), Tregs (C) of PSO at the level of CLA+, and MAITs (D, E), γδ T cells (F, G) of PSO at the levels of Cluster and CLA+. [file Image_4.jpeg]

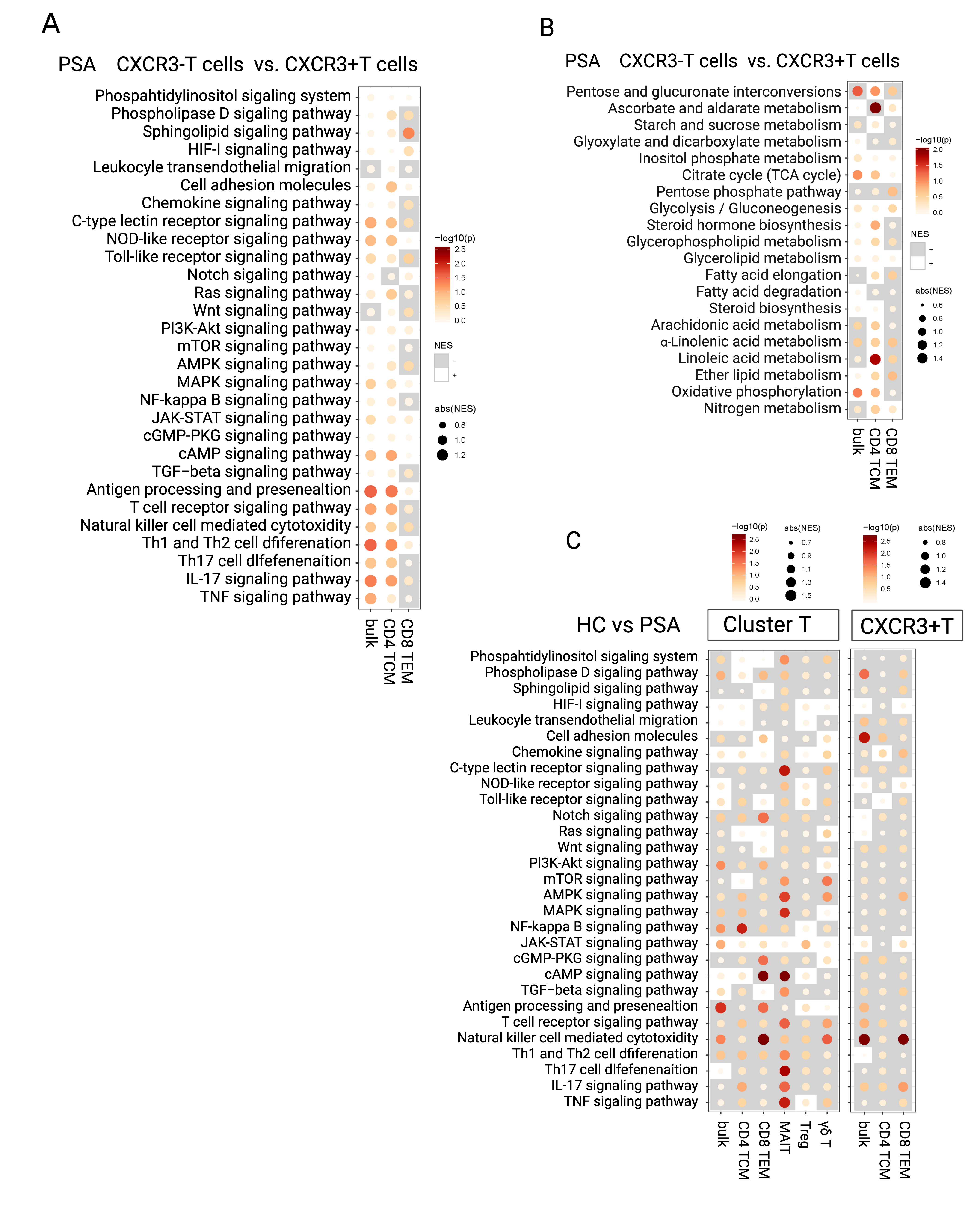

Supplement: Supplementary Figure 5 — Immunometabolic difference landscape of T cells within PSA, and between PSA and HC. (A, B) Immunometabolic difference landscape between subfractions of CXCR3-T cells and CXCR3+T cells in PSA by GSEA. (C) Immune signaling difference landscape of focused subsets between PSA and HC at the levels of Cluster and CXCR3+ by GSEA. Gray background indicates a negative normalized enrichment score (NES) and the bright background indicates a positive NES. The NES reflects the degree to which a gene set is downregulated (negative NES) or upregulated (positive NES). The area of the dot corresponds to the NES absolute value. The color of the dots corresponds to the P value. [file Image_5.jpeg]

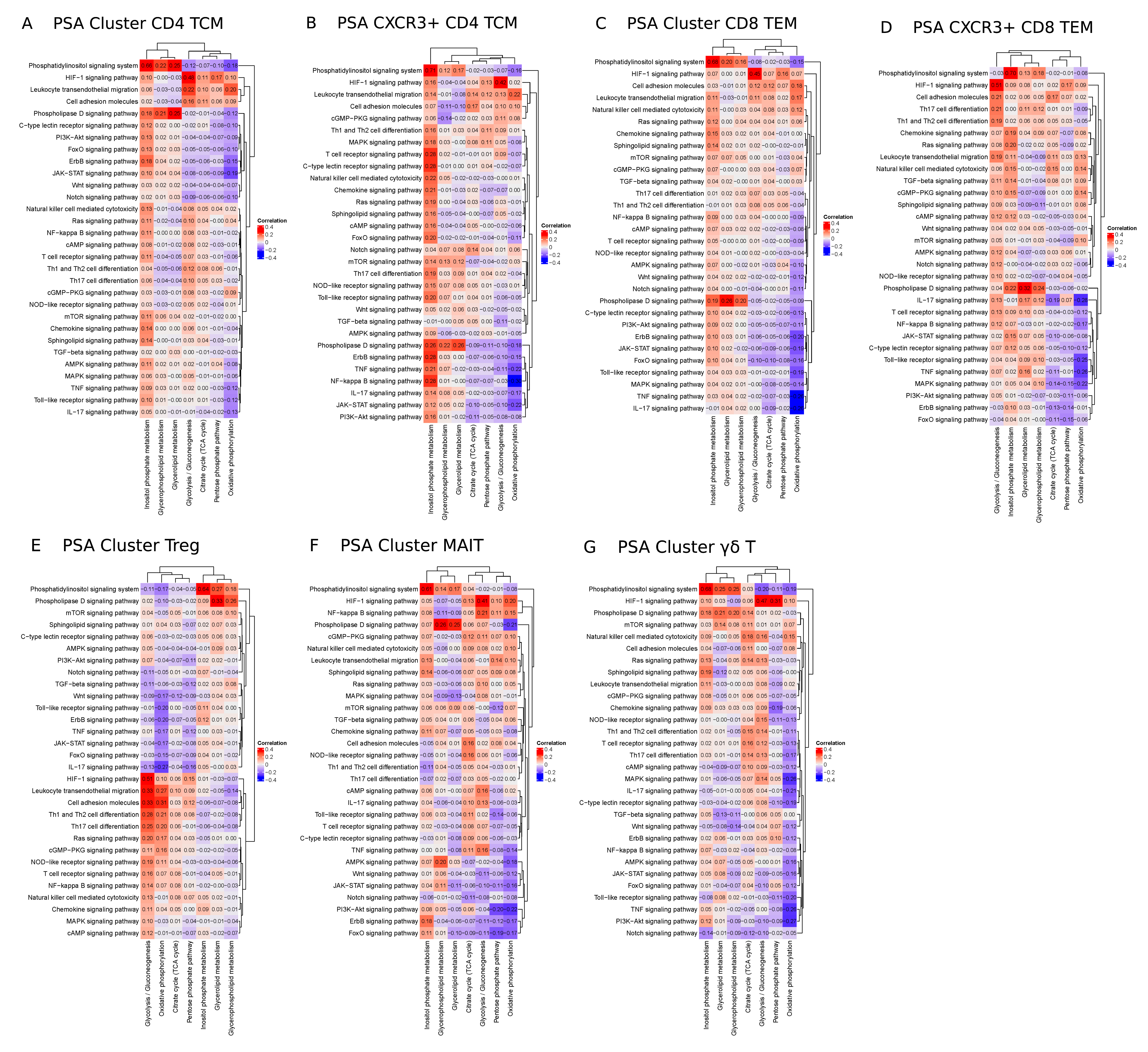

Supplement: Supplementary Figure 6 — Immunometabolic linkage between metabolic pathways of inositol phosphate metabolism, glycerolipid metabolism, glycerophospholipid metabolism, glycolysis/gluconeogenesis, pentose phosphate pathway, citrate cycle (TCA cycle), or oxidative phosphorylation and immune events in CD4+ TCMs (A, B), CD8+ TEMs (C, D) of PSA at the levels of Cluster and CXCR3+, and Tregs (E), MAITs (F), γδ T cells (G) of PSA at the level of Cluster. [file Image_6.jpeg]

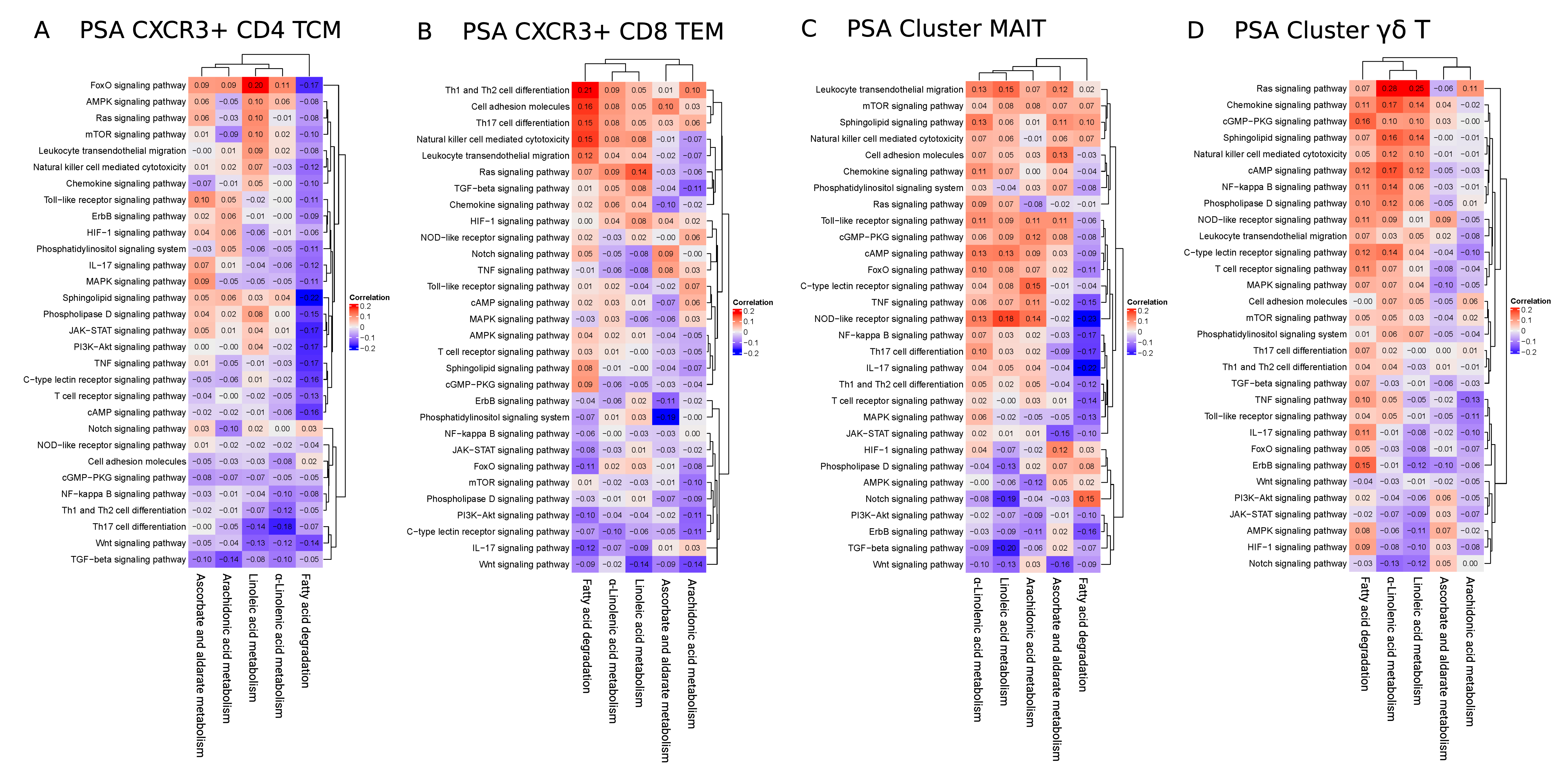

Supplement: Supplementary Figure 7 — Immunometabolic lingkage between metabolic pathways of α-linolenic acid metabolism, linoleic acid metabolism, arachidonic acid metabolism, ascorbate and aldarate metabolism, or fatty-acid degradation and immune events in T cells of PSA. Immunometabolic lingkage in CD4+ TCMs (A) and CD8+ TEMs (B) of PSA at the level of CXCR3+, and MAITs (C), γδ T cells (D) of PSA at the level of Cluster. [file Image_7.jpeg]
