# Supplementary material for: Single-cell transcriptomic landscape of immunometabolism reveals intervention candidates of ascorbate and aldarate metabolism, fatty-acid degradation and PUFA metabolism of T-cell subsets in healthy controls, psoriasis and psoriatic arthritis
Source: Front Immunol. 2023 Jul 10;14:1179877. doi: 10.3389/fimmu.2023.1179877 (PMC10363747; doi:10.3389/fimmu.2023.1179877)
Supplement: Supplementary file 8 [file Table_1.docx]

| **Supplementary Table S1.** Information and grouping of subjects. | | | | | | | | | |
| --- | --- | --- | --- | --- | --- | --- | --- | --- | --- |
|  | | | | | | | | | |
| Specimen | Age | Gender | Status | Psoriasis BSA or (PASI) | Joint pain | Systemic Medications | Group level 1 | Group level 2 | Group level 3 |
| HC01 | 61 | M | Healthy | NA | N | None | HC |  |  |
| HC02 | 64 | M | Healthy | NA | N | None | HC |  |  |
| HC03 | 37 | F | Healthy | NA | N | None | HC |  |  |
| HC04 | 28 | M | Healthy | NA | N | None | HC |  |  |
| HC05 | 26 | F | Healthy | NA | N | None | HC |  |  |
| HC06 | 72 | F | Healthy | NA | N | None | HC |  |  |
| HC07 | 41 | F | Healthy | NA | N | None | HC |  |  |
| HC08 | 57 | F | Healthy | NA | N | None | HC |  |  |
| HC09 | 49 | F | Healthy | NA | N | None | HC |  |  |
| HC10 | 38 | M | Healthy | NA | N | None | HC |  |  |
| HC11 | 59 | M | Healthy | NA | N | None | HC |  |  |
| HC12 | 35 | M | Healthy | NA | N | None | HC |  |  |
| HC13 | 37 | F | Healthy | NA | N | None | HC |  |  |
| HC14 | 26 | M | Healthy | NA | N | None | HC |  |  |
| HC15 | 22 | F | Healthy | NA | N | None | HC |  |  |
| HC16 | 26 | F | Healthy | NA | N | None | HC |  |  |
| HC17 | 32 | M | Healthy | NA | N | None | HC |  |  |
| HC18 | 47 | M | Healthy | NA | N | None | HC |  |  |
| HC19 | 41 | F | Healthy | NA | N | None | HC |  |  |
| HC20 | 48 | F | Healthy | NA | N | None | HC |  |  |
| HC21 | 64 | M | Healthy | NA | N | None | HC |  |  |
| HC22 | 34 | F | Healthy | NA | N | None | HC |  |  |
| HC23 | 28 | M | Healthy | NA | N | None | HC |  |  |
| HC24 | 25 | F | Healthy | NA | N | None | HC |  |  |
| HC25 | 36 | F | Healthy | NA | N | None | HC |  |  |
| HC26 | 33 | M | Healthy | NA | N | None | HC |  |  |
| HC27 | 46 | F | Healthy | NA | N | None | HC |  |  |
| HC28 | 48 | F | Healthy | NA | N | None | HC |  |  |
| HC29 | 52 | F | Healthy | NA | N | None | HC |  |  |
| PSO01 | 39 | M | PSO | NA | N | None | PSO |  |  |
| PSO03 | 79 | F | PSO | (60) | N | None | PSO |  |  |
| PSO04 | 32 | M | PSO | (7) | Y | None | PSO |  |  |
| PSO06 | 53 | F | PSO | (31.1) | Y | None | PSO |  |  |
| PSO07 | 57 | M | PSO | (7.8) | N | None | PSO |  |  |
| PSO08 | 28 | F | PSO | (13.2) | N | None | PSO |  |  |
| PSO09 | 28 | M | PSO | (19.8) | Y | None | PSO |  |  |
| PSO11 | 48 | F | PSO | 5 | N | None | PSO |  |  |
| PSO13 | 31 | M | PSO | 4 | N | None | PSO |  |  |

| Specimen | Age | Gender | Status | Psoriasis BSA or (PASI) | Joint pain | Systemic Medications | Group level 1 | Group level 2 | Group level 3 |
| --- | --- | --- | --- | --- | --- | --- | --- | --- | --- |
| PSO14 | 66 | F | PSO | 3 | N | None | PSO |  |  |
| PSO16 | 29 | F | PSO | 3 | N | None | PSO |  |  |
| PSO17 | 33 | F | PSO | 4 | N | None | PSO |  |  |
| PSO18 | 58 | F | PSO | 3 | N | None | PSO |  |  |
| PSO21 | 53 | M | PSO | 4 | N | None | PSO |  |  |
| PSO22 | 38 | M | PSO | 4 | N | None | PSO |  |  |
| PSO24 | 37 | M | PSO | 4 | N | None | PSO |  |  |
| PSA01 | 36 | F | PSA | 2 | Y | methotrexate | PSA_SM | MTX |  |
| PSA05 | 21 | M | PSA | 2 | Y | brodalumab | PSA_SM | PSA_Anti | Brodalumab |
| PSA06 | 35 | M | PSA | 3 | Y | secukinumab | PSA_SM | PSA_Anti | Secukinumab |
| PSA07 | 37 | M | PSA | 1 | Y | adalimumab | PSA_SM | PSA_Anti | Adalimumab |
| PSA09 | 38 | M | PSA | 4 | Y | adalimumab | PSA_SM | PSA_Anti | Adalimumab |
| PSA11 | 60 | M | PSA | 2 | Y | methotrexate | PSA_SM | MTX |  |
| PSA12 | 51 | M | PSA | 2 | Y | None | PSA |  |  |
| PSA13 | 35 | F | PSA | 5 | Y | adalimumab | PSA_SM | PSA_Anti | Adalimumab |
| PSA16 | 31 | F | PSA | 5 | Y | None | PSA |  |  |
| PSA17 | 36 | F | PSA | 1 | Y | risankizumab | PSA_SM | PSA_Anti | Risankizumab |
| PSA18 | 73 | F | PSA | 5 | Y | None | PSA |  |  |
| PSA19 | 51 | F | PSA | 5 | Y | None | PSA |  |  |
| PSA20 | 32 | M | PSA | 5 | Y | adalimumab | PSA_SM | PSA_Anti | Adalimumab |
| PSA21 | 37 | M | PSA | 1 | Y | etanercept | PSA_SM | PSA_Anti | Etanercept |
| PSA22 | 32 | M | PSA | 1 | Y | adalimumab | PSA_SM | PSA_Anti | Adalimumab |
| PSA23 | 49 | M | PSA | 4 | Y | secukinumab | PSA_SM | PSA_Anti | Secukinumab |
| PSA24 | 54 | M | PSA | 3 | N | None | PSA |  |  |
| PSA26 | 77 | M | PSA | 3 | Y | etanercept | PSA_SM | PSA_Anti | Etanercept |
| PSA27 | 33 | F | PSA | 2 | Y | None | PSA |  |  |
